# Time-driven activity-based cost of outpatient total hip and knee arthroplasty in different set-ups

**DOI:** 10.1080/17453674.2018.1496309

**Published:** 2018-08-06

**Authors:** Henrik Husted, Billy B Kristensen, Signe E Andreasen, Christian Skovgaard Nielsen, Anders Troelsen, Kirill Gromov

**Affiliations:** 1 Department of Orthopedic Surgery, Copenhagen University Hospital Hvidovre, Copenhagen;; 2 Ambulatory Surgery Department, Copenhagen University Hospital Hvidovre, Copenhagen, Denmark

## Abstract

Background and purpose — Length of stay (LOS) following total hip and knee arthroplasty (THA and TKA) has been reduced over the years due to fast-track. Short stays of 2 days in fast-track departments in Denmark have resulted in low total costs of around US$2,550. Outpatient THA and TKA is gaining popularity, albeit in a limited and selected group of patients; however, the financial benefit of outpatient arthroplasty remains unknown. We present baseline detailed economic calculations of outpatient THA and TKA in 2 different settings: one from the hospital and another from the ambulatory surgery department.

Patients and methods — Data from 6 patients (1 TKA, 1 uncemented THA, 1 cemented THA in each department) were collected prospectively using the Time Driven Activity Based Costing method (TDABC). Time consumed by different staff members involved in patient treatment in the perioperative period of outpatient THA and TKA was calculated in 2 different settings: one in the orthopedic department and one in the ambulatory surgery department.

Results — LOS was around 11 h in the orthopedic department and around 7 h in the ambulatory surgery department, respectively. TDABC revealed minor differences in the operative settings between departments and similar expenses occurred during the short stay of US$777 and US$746, respectively. Adding the preoperative preparation and postoperative follow-up resulted in total cost of US$951 and US$942 for the ward and the ambulatory surgery department, respectively.

Interpretation — Outpatient THA and TKA in hospital and ambulatory surgery departments results in similar cost using the TDABC method. Compared with the cost associated with 2-day stays, outpatient procedures are around two-thirds cheaper provided no increase occurs in complications or readmissions.

Length of stay (LOS) in hospital following total hip and knee arthroplasty (THA and TKA) has been reduced over the years. In particular, fast-track THA and TKA combining evidence-based clinical features with organizational optimization has resulted in short LOS of 1–3 days for the majority of unselected patients as a positive spin-off from the main goal of reducing perioperative morbidity and mortality (Husted et al. 2010, 2016, Malviya et al. 2011, Husted 2012, Jørgensen et al. 2013a and b, Khan et al. 2014).

In the last decade, reports of patients operated with THA and TKA being discharged even faster have been published (Berger et al. 2009a, 2009b). However, concern has been expressed regarding the cost-benefit of this approach as the additional services provided by the additional personnel may outweigh the savings of a shorter stay (Berger et al. 2009b). Since then, several reports have shown the feasibility of outpatient arthroplasty in selected patients (Hartog et al. 2015, Goyal et al. 2017, Gromov et al. 2017, Klein et al. 2017, Larsen et al. 2017, Meneghini et al. 2017).

Conventional inpatient stays have recently been reported to cost around US$30,000 in the US (Nichols and Vose 2016) and with expensive reimbursement at around US$26,000 on average in a Medicare population (Mechanic 2015). Fast-track THA and TKA with a 2-day stay was calculated at around US$2,500 in Denmark for comparison using the Time Driven Activity Based Costing method (TDABC), which represents an economical method using only staff-based costs of the procedures in the care-cycle (Andreasen et al. 2017).

A few studies have used various methods to determine the potential economic benefit of outpatient arthroplasty (Aynardi et al. 2014, Lovald et al. 2014, Huang et al. 2017) but their different set-ups including non-itemized billing, overhead and indirect costs, and 2 years’ compiled expense hinder comparison between hospitals as different reimbursement systems are used.

As several editorials have been addressing the need for detailed economic evaluation of the outpatient procedure itself (Argenson et al. 2016, Vehmeijer et al. 2018), and reimbursement systems vary between hospitals, we found it of interest to use the TDABC method to calculate the cost for the outpatient procedure in different outpatient set-ups allowing for comparison between hospitals and countries, regardless of reimbursement system.

Thus, we present baseline detailed economical calculations using TDABC of outpatient THA and TKA in a hospital and an ambulatory surgery department.

## Methods

### Economic considerations

Time-driven activity-based costing (TDABC) (Kaplan and Anderson 2004) is a method to calculate cost involving only estimates of 2 parameters: the time needed from staff members to perform a process or activity and the cost per minute of that staff member. Combining the different processes and the staff members involved, TDABC will reflect time and cost spent on operational processes in detail. The TDABC takes into account the amount of time spent by various staff members on patient-related activities only, and the overhead value of staff-time spend not directly with patients or patient-related work (holidays, illness, breaks, research, and education) is neglected.

Prerequisites for the estimation of TDABC are the creation of process maps in the care cycle, a calculation of time spent on the various specific processes and knowing the salaries of the staff members involved.

Adding other expenses in the care cycle for THA and TKA related to consumables, surgery (exclusive of implants), utensils, medicine, tests, and cleaning gives the total cost for the specific procedure. However, these expenses are not calculated or included here as they are not part of the TDABC method and may vary considerably between institutions. Also, fixed cost like buildings, equipment in surgery and rooms, heating and lighting, and administration will not be taken into account. By looking only at the direct costs, the comparison of cost incurred by different departments will be possible across potential differences in organizational set -up.

The care cycle of a total joint arthroplasty (TKA) (THA or TKA) is described in 2 different settings: 1 in the orthopedic department and 1 in the ambulatory surgery department (Figure). All procedures involving staff members were evaluated and timed: Time related to surgical procedures and anesthesia was collected from the surgical database, which gives exact time spent including detailed information on every procedure in the operating room. Other procedures were noted by both the staff members and by an independent observer to ensure completeness. Preoperative procedures differed slightly whereas the follow-up procedures were identical. A full care cycle is defined from the first preoperative visit to the final outpatient follow-up. All patients were seen by a nurse at an outpatient clinic at 2 (THA) or 3 (TKA) weeks postoperatively for staple removal. All patients were seen by the surgeon at 3 months postoperatively. Time spent on these procedures as for the preoperative procedures is an estimate based on the time slots and mean time spent on each patient in group settings.

To allow for the same case mix, 1 operation with a hybrid THA (cemented femur), 1 operation with an uncemented THA, and 1 operation with a cemented tricompartmental TKA were timed in each setting and a “typical” course was ensured (no abnormal procedures regarding logistics or clinical procedures (anesthesia, surgery). Patients were not informed about nor consented to timing the various procedures as timing did not influence any procedure or content thereof and the 6 patients were chosen for type of surgery on days of timing. No randomization of patients or location of surgery was performed and patients were already scheduled for surgery on the 2 different lists. There was no choosing of a specific patient for the specific location by any factor except vacancy. Accurate times were registered and mean time for all operative procedures was calculated as there were slight differences (Table 3). Information on salaries was obtained from the central hospital database and represents the average salary for each staff type (Table 1). Based on these calculations, TDABC was estimated for an operation in both settings.

**Table 1.** Cost per minute in US$for various staff members involved

Staff members	CPM
(Hvidovre)	US$
Orthopedic surgeon	2.00
Anesthesiologist	1.33
Orthopedic resident	1.20
Nurse (ward)	0.85
Nurse (anesthesia)	0.94
Nurse (scrub, OR)	0.84
Nurse assistant	0.75
Physiotherapist	0.88
Cleaning	0.75
Radiologist	2.00
Secretary	0.75
Laboratory technician	0.85
Porter	0.64

### Treatment procedure

All patients scheduled for outpatient THA or TKA were screened for eligibility (Gromov et al. 2017). Eligible patients were included and informed of the intended outpatient procedure. The surgery was performed under spinal analgesia in the OR and under general anesthesia in the ambulatory surgery department. THA was performed using a standard posterolateral approach with simple posterior soft-tissue repair and TKA was performed with a standard medial parapatellar approach without the use of tourniquet; LIA was given at the end of surgery. Postoperative radiographs were obtained in the OR in the ambulatory surgery department (in the radiographic department for the hospital patients), approved by the surgeon and given to the patient. Rivaroxaban was used as oral thromboprophylaxis starting 6 to 8 hours postoperatively and given for 2 days only. Mechanical thromboprophylaxis and extended oral thromboprophylaxis was not used. In Denmark the recommendations allow for oral thromboprophylaxis only during hospital stay if the patient is following a fast-track pathway (Jørgensen et al. 201b).

Patients in hospital bypass the post-anesthesia care unit (PACU) if blood loss is estimated less than 300 mL in order to begin their functional recovery as early as possible—no patient in this study stayed in the PACU.

Physiotherapy was started as soon as possible after surgery: after the spinal anesthesia had worn off or when patients were fit to mobilize. Patients were discharged if fulfilling the discharge criteria before 8 pm. The discharge criteria were: self-dependent, sit/stand from chair/toilet, steady gait with crutches, master stairs, stable vital signs, acceptable pain (VAS <3 at rest and VAS <5 on mobilization), postsurgical bleeding should be consistent with expected blood loss for the procedure and not require repeated dressing change, hemodynamically stable and showing no clinical signs of anemia (Husted 2012). All patients were discharged to their own homes without any additional assistance.

Pre- and postoperative outpatient visits were the same without differences in duration, except for the preoperative patient seminar, which was exclusively for the hospital patients, whereas the patients to be operated in the ambulatory surgery center had slightly longer preoperative preparation by the anesthetist. Patients operated in the ambulatory surgery department also received preoperative physiotherapy instructions, while physiotherapy was covered during the patient seminar for hospital patients.

### Statistics

Descriptive statistics is used reporting minutes spent on processes and activities and cost in US$.

### Ethics, funding, and potential conflict of interest

No approval from the National Ethics Committee was necessary as this was a non-interventional observational study. Permission to store and review patient data was given by the Danish National Board of Health (j.nr:3-3013-56/1/HKR) and Danish Data Protection Agency (j.nr:20047-58-0015). No competing interests were declared.

## Results

All 6 patients fulfilled the discharge criteria on the day of surgery and were discharged. Patients on the ward stayed till 6 pm, whereas patients in the ambulatory surgery department stayed till 3 pm. Time used during the entire care cycle, and corresponding cost, is presented in Table 2.

**Table 2.** Time (minutes) and corresponding TDABC (US$) used on preoperative preparation before operations in ambulatory surgery department (ASD) and central operation ward (COP) and post-discharge follow-up

Perioperative activities	ASD		COP	
	time	TDABC	time	TDABC
	(min)	(US$)	(min)	(US$)
Preop. visit 1:				
Surgeon	20	40	20	40
Total	20	40	20	40
Preop. visit 2:				
Resident	30	36	30	36
Nurse	30	26	30	26
Lab technician	10	9	10	9
Anesthetist	20	27	10	13
Physiotherapist	15	13	X	0
Total	105	110	80	83
Preop. visit 3 (patient seminar, n = 30):				
Surgeon	X		30	60
Anesthetist nurse	X		15	14
Physiotherapist	X		15	13
Nurse assistant	X		90	68
Total	0		150	155
Day of surgery:				
Total	671	746	701	777
Follow-up (nurse):				
Nurse	30	26	30	26
Total	30	26	30	26
Follow-up (surgeon):				
Surgeon	10	20	10	20
Total	10	20	10	20
Total	836	942	846	951

Time used by staff members for the various procedures during the day of surgery is listed in Table 3 as is the TDABC for each procedure per staff member. Time for the different procedures varied a little between operations and between locations, reflecting the small variabilities even in a standard procedure: they accumulated to 701 minutes in total for the hospital patients versus 671 minutes for the ambulatory surgery department patients (Table 3). Mean cost for the 3 operations was US$777 on the ward and US$746 in the ambulatory surgery department (Table 3). Adding the time spent on preoperative preparation and postoperative follow-up, a total of 846 versus 836 minutes were accumulated amounting to US$951 and US$942, respectively (Table 3).

**Table 3.** Time (minutes) and corresponding TDABC (US$) used on various procedures in 3 operations (TKA = total knee arthroplasty, THAh = total hip arthroplasty hybrid, THAu = total hip arthroplasty uncemented) in ambulatory surgery department (ASD) and central operation ward (COP) during day of surgery

	ASD1	ASD2	ASD3	time	TDABC	COP1	COP2	COP3	time	TDABC
	TKA	THAh	THAu	mean	US$	TKA	THAh	THAu	mean	US$
Anesthetist:										
Anesthesia procedure	13	11	8			15	8	16		
Total	13	11	8	11	15	15	8	16	13	17
Anesthetist nurse:										
Anesthesia prep. (+ iv lines)	15	13	8			35	35	10		
Anesthesia procedure	13	11	9			15	10	10		
Surgery	35	57	45			43	48	39		
Postoperative tasks	5	5	5			5	5	9		
Total	68	86	67	74	70	98	98	68	88	83
Surgeon:										
Preparation outside OR	8	10	10			10	10	10		
Preparation inside OR	28	20	22			35	25	27		
Scrubbing	5	5	5			5	5	5		
Surgery	35	57	45			43	48	39		
Postop. radiographs	8	6	7			X	X	X		
Documentation	10	10	11			5	5	5		
Rounds	15	12	14			15	15	15		
Total	109	120	114	114	228	113	108	101	107	214
Surgical assistant:										
Preparing prostheses, bed	15	20	16			20	10	10		
Preparing op table/positioning	15	13	15			23	15	13		
Scrubbing	5	5	5			5	5	5		
Surgery	35	57	45			43	48	39		
Postop. radiographs	8	6	7			X	X	X		
Taking patient to recovery room	10	8	10			13	15	15		
Total	88	109	98	98	118	104	93	82	93	112
Scrub nurse:										
Preparation OR	15	15	15			10	15	15		
Scrubbing	5	5	5			5	10	5		
Prep. instruments	9	10	15			10	20	7		
Anesthesia procedure	13	11	12			15	8	16		
Sterilization, draping	17	16	16			13	20	10		
Surgery	35	57	45			43	48	39		
Cleaning instruments	16	10	15			13	10	10		
Total	114	124	123	120	101	109	131	102	114	96
Floor nurse:										
Preparation OR	11	15	15			10	15	12		
Prep. instruments	17	15	20			20	10	7		
Anesthesia procedure	13	11	13			15	8	16		
Sterilization, draping	17	15	15			13	20	10		
Surgery	35	57	45			43	48	39		
Postop. radiographs	8	8	8			X	X	X		
Cleaning	10	10	15			7	5	10		
Total	111	131	131	124	104	108	106	94	103	87
Radiologist:										
Postop radiographs	X	X	X			10	10	10		
Total	0	0	0	0	0	10	10	10	10	20
Ward nurse:										
Prep. of patient	5	5	5			22	15	10		
Various incl. documentation	90	92	92			102	100	80		
Total	95	97	97	96	82	124	115	90	110	94
Physiotherapist:										
Mobilization	30	35	35			60	60	70		
Total	30	35	35	33	29	60	60	70	63	55
Total staff minutes	628	713	673	671	746	741	729	633	701	777

## Discussion

In this prospective study we provide baseline detailed economic calculations using TDABC of outpatient THA and TKA in a hospital ward and in an ambulatory surgery department.

The time and money spent to do an outpatient THA or TKA varied only slightly between the hospital ward and the ambulatory surgery department (situated separately in the hospital). Slightly different pathways and procedures were followed resulting in a total of around 14 hours of staff time used per procedure resulting in a difference of only US$9 in total. The amounts of US$951 and US$942 are of course significantly lower compared with the previously calculated US$2,550 for a 2-day fast-track stay (Andreasen et al. 2017), which is mostly attributable to the extra nights the patient spent in the hospital and the associated care.

All 6 patients in this study were discharged on the day of surgery. However, feasibility studies have shown discharge on the day of surgery (defined as intended within 12 h) to vary between 30% and 89% (Hartog et al. 2015, Goyal et al. 2017, Gromov et al. 2017, Larsen et al. 2017). With no study reaching 100%, it is evident that precautions in the form of the possibility for an extended stay overnight are mandatory. The pathophysiological changes associated with the surgical stress response pose barriers to fulfilling functional discharge criteria early (i.e., on the day of surgery) and as these include pain, dizziness (orthostatic intolerance), and muscle weakness, a multimodal and multidisciplinary effort is needed but not successfully accomplished in all patients (Husted et al. 2011). Logistically, bypassing the PACU or having very short stays there may require a reevaluation of discharge criteria from the PACU to facilitate outpatient arthroplasty (Lunn et al. 2012). Occasionally, patients intended to be outpatients have to stay in hospital/be admitted to hospital. This is easily facilitated in a hospital ward where the bed is available, at least till next day depending on the booking of beds, and as nurses are present already no extra resources need be allocated. In the ambulatory surgery department (in our hospital), no imminent possibility for an overnight stay is present and hence this requires either staff to stay over or referral to a 24 h-manned ward, both requiring extra resources inflicting costs that are not accounted for in the presented TDABC. Hence, there are some potential hidden costs in performing outpatient arthroplasty not accounted for by the TDABC, which may actually render outpatient arthroplasty in the hospital ward cheaper, of course depending on the proportion of subsequent overnight stays, as the subsequent number of patients needing an inpatient stay may be “absorbed” in the (already existing) ward nurse manning. Also, the price for an “empty bed” for those patients operated in the hospital setting is not accounted for in the TDABC, which thus also presents a potential hidden cost. Unfortunately, making estimations for such hidden costs in a TDABC is virtually impossible, as this would require many assumptions and potentially inaccurate estimations that would make the final result difficult to interpret and hinder comparison with other hospital and department set-ups.

TDABC analysis does not include overhead expenses. While these costs can be substantial, including overheads may make comparison with other hospitals even more inaccurate as overhead expenses and their composition may vary greatly between the private and the public sector and even between hospitals within the same country.

Selection criteria for outpatient arthroplasty as well as safety issues have been addressed mainly in large cohorts like the American College of Surgeons–National Surgical Quality Improvement Program (NSQIP), but unfortunately the data in the cohorts are blinded and rely on the hospitals’ definition of the outpatient procedure. As a study has shown this definition to be severely flawed by also including patients staying in hospital for “observation” for 1 or more nights (Bovonratwet et al. 2017)—and still counted as outpatients—the outcomes of the studies using NSQIP data on patient selection and safety should be interpreted with caution. Also, in some studies examining outpatient arthroplasty and looking at safety, the treatment continues after discharge as the hospital is “moved home with the patient” as both nurses and physiotherapists visit and treat the patient at home (Klein et al. 2017). Hence, valid data are lacking on safety in larger groups of patients and also hindering economic cost–benefit evaluation of the true outpatient procedure with same-day discharge to home without nurses or physiotherapists visiting including 90-day complication/readmission rates in larger groups. Hence, safety issues are still pending regarding outpatient versus inpatient arthroplasty and might add to cost, if more complications/readmissions were to occur (Lovecchio et al. 2016). The current publications, either finding it safe (Courtney et al. 2017, Nelson et al. 2017, Courtney et al. 2018) or associated with more complications (Lovecchio et al. 2016, Arshi et al. 2017) may not answer the question of safety as they are based on register studies with poor or no control of the definition of “outpatient” (Bovonratwet et al. 2017). Also, reviews call for prospective cohort studies to determine outcome, safety, and cost efficiency of outpatient arthroplasty (Pollock et al. 2016, Hoffmann et al. 2018). If extra resources are needed with the sole purpose of allowing discharge on the day of surgery, cost–benefit analyses need to be performed including perioperative safety (Argenson et al. 2016, Thienpont et al. 2015, Vehmeijer et al. 2018).

We have not focused on potential complications, readmissions, or mortality in this study as the aim was to evaluate a care cycle for the standard arthroplasty outpatient. As the aim was to illuminate the staff-associated time and the cost thereof in outpatient arthroplasty—and not to compare the 2 pathways to find the cheapest—only 6 patients were included to allow calculations for the various procedures. No power calculation was performed or needed as description and calculation of the procedures allowing for comparison was intended.

No other studies using the TDABC method have been published for outpatient hip and knee arthroplasty. Other accounting methods exist and TDABC may underestimate the total cost by the inherent weaknesses addressed above and by not taking overheads, heating, linen, food, indirect costs etc. into account. However, the TDABC method is still recommended as it provides a care cycle-based identification of care processes and the associated staff minutes allowing for evaluation, comparison, and optimization (Palsis et al. 2018). Hence, our study provides a preliminary baseline for comparison with longer hospital stays (Akhavan et al. 2016, Andreasen et al. 2017) as well as more conventional tracks (Chen et al. 2015). When larger prospective cohort studies focusing on morbidity and mortality in outpatients are published, the figures need to be included in more detailed economic analyses allowing for a more accurate cost–benefit analysis.

It can and should be debated whether TDABC is the best method to perform a detailed economic evaluation or if other more detailed analyses are warranted, i.e. the Patient and Family Centered Care Methodology and Practice (PFCCM/P). This hybrid process map includes TDABC plus “behind-the-scene activities” such as central sterile processing and billing, non-direct personnel time, and patient and family waiting time (DiGioia et al. 2016). However, we find the TDABC method reliable, reproducible, simple, and transparent to allow for comparison and strategic optimization of care processes. In a recent study from the US comparing TDABC for TKA in 29 hospitals, total personnel cost varied by a factor of 2.3 with nursing costs and length of stay being some of the more expensive drivers (Haas and Kaplan 2016). Discharge destination was another driver for total cost and hence the study supports evaluation of the logistical set-up, staff use, short stay, and discharge to home if feasible. The TDABC method is not without bias. Performance bias could occur as staff know time measurement is taking place and also external validity may be questioned as the 6 measured operations all represent flawless pathways without delays of any kind.

As the demand for THA and TKA is increasing in a financially challenged environment and an increasing number of procedures are likely to be performed on an outpatient basis, it is important to perform evaluation of pathways, of the staff minutes used, and of the economic expenses associated herewith.

HH, BBK, SEA, and KG conceived the study; all authors collected data and evaluated them. HH wrote the first draft of the manuscript, and all authors revised it and approved the final version to be published.

*Acta* thanks Ola Rolfson and other anonymous reviewers for help with peer review of this study.

**Figure:**
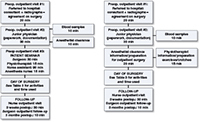
Figure: Flowchart of various procedures before, during, and after outpatient TJA in a central operation ward (left) and an ambulatory surgery department (right)
